# Protective Effect of Palmitoleic Acid on Alcoholic Fatty Liver Injury in Mice: Insights from Gut Microbiota and Untargeted Metabolomics Analysis

**DOI:** 10.3390/nu18213536

**Published:** 2026-10-27

**Authors:** Yu Liu, Benkang Su, Minghui Yang, Bingqing Shang, Yue Geng, Qiu Wu

**Affiliations:** College of Life Sciences, Shandong Normal University, Ji’nan 250358, China2025318137@stu.sdnu.edu.cn (B.S.);

**Keywords:** POA, alcoholic fatty liver disease, microbiota, metabolomics

## Abstract

Background: Alcoholic fatty liver disease (AFLD) represents an early, reversible stage within the spectrum of alcoholic liver disease. Palmitoleic acid (POA), classified as an ω-7 MUFA, has previously demonstrated hepatoprotective properties against non-alcoholic fatty liver disease, but the protective effect of it in AFLD and its impact on the intestinal flora and metabolites remain unclear. The research sought to elucidate the protective role of POA in AFLD in a murine model and corresponding mechanistic pathways. Methods: Thirty male Kunming mice were randomly distributed across five experimental cohorts: a healthy control group, an alcohol-exposed model group, and three POA-treated groups receiving daily different doses. With the exception of the control cohort, all animals underwent intragastric administration of 53% ethanol once daily. Multiple endpoints were evaluated, including hepatic function markers, lipid metabolic profiles, antioxidant capacity, liver histopathological features, colonic microbial composition, and intestinal metabolomic signatures. Results: Our findings revealed that POA supplementation markedly lowered hepatic and serum concentrations of ALT, AST, TBA, AKP, TBIL, TP, Alb, and MDA, while it concurrently returned HDL-C and TG levels, thereby effectively counteracting alcohol-triggered hepatic injury. Moreover, POA mitigated alcohol-associated gut microbial imbalance by decreasing *Firmicutes*/*Bacteroidetes* and remodeled the relative abundances of *Turicibacter*, *Prevotellaceae UCG-001*, *Akkermansia* and *Bacteroides*. Metabolomic profiling further demonstrated that POA normalized perturbed levels of Hispidol, Suberosin, Val-Trp-His, and *N*-acetylserine and influenced metabolic pathways related to digestion, lipid processing, and amino acid turnover. Correlation analyses validated robust interrelationships among key differential bacterial taxa, altered metabolites, and hepatic injury biomarkers in AFLD mice. Conclusions: In conclusion, POA exerts a protective effect against AFLD in mice, and this hepatoprotective effect is associated with the amelioration of gut dysbiosis and the restoration of intestinal metabolic profiles. These findings provide preliminary scientific evidence for the development of POA as a functional food ingredient for AFLD prevention and control.

## 1. Introduction

As a typical member of ω-7 MUFAs, palmitoleic acid (POA) exhibits poor solubility in aqueous media but dissolves readily in alkaline solutions and various organic solvents [1]. Natural sources rich in POA include cod liver, macadamia, sea buckthorn from Shanxi province, and oils derived from deep-sea fish [2,3]. In recent years, the Cell Metabolism, Annals of Internal Medicine and Diabetes have successively reported that POA can improve insulin sensitivity, regulate lipid metabolism, reduce inflammatory responses and alleviate liver lipid toxicity [4,5,6]. Research has indicated that POA exerts an influence on glucose homeostasis, insulin resistance, and diabetes [7]. Furthermore, emerging evidence supports the preventive and alleviating actions of POA on non-alcoholic fatty liver disease [8]. Underlying mechanisms involve AMPK pathway activation, enhanced fat oxidation, and downregulated expression of SREBP1 and SCD1 and FAS, ultimately leading to diminished hepatic lipid accumulation [9,10]. Therefore, we speculate that POA may also have preventive and alleviating effects on alcoholic fatty liver disease (AFLD).

Alcoholic liver disease (ALD) is a prominent global public health concern, posing a severe threat to individuals’ well-being [11]. On a global scale, approximately 2.3 billion individuals consume alcoholic beverages [12]. Among this population, approximately 23.6 million individuals are afflicted with compensated alcoholic cirrhosis, and an additional 2.5 million are affected by decompensated alcoholic cirrhosis [13]. Chronic alcohol misuse can lead to irreversible impairment of human organ functions and even death [14]. AFLD is the early manifestation of ALD and can advance to more serious pathological conditions, including liver cirrhosis, hepatic fibrosis, and even hepatocellular carcinoma (HCC) [15,16,17]. Therefore, we hope to develop a natural and healthy liver-protecting food.

POA has been preliminarily demonstrated to improve insulin sensitivity, regulate lipid metabolism, and alleviate liver lipid toxicity [4,5,6]. AFLD also exhibits various internal symptom changes, such as intestinal flora imbalance [18]. Therefore, the animal experiments have been designed, and the experimental mice were administered 53% alcohol and POA. Liver function indicators, serum lipid metabolism levels, and antioxidant capacity were detected. Subsequently, identification of colonic content metabolomic profiles and employment of 16S rRNA high-throughput sequencing for colonic microbiota structural analysis. Analysis of the association among significant liver injury, metabolites, and intestinal microbiome community for exploration of the protective mechanism of POA in AFLD mice.

## 2. Materials and Methods

### 2.1. Materials and Chemicals

Palmitoleic acid, 53% ethanol solution, and 0.9% normal saline were bought from Macklin Co., Ltd. (Beijing, China). Assay kits for the following biochemical markers were bought from the Nanjing Jiancheng Bioengineering Institute (Nanjing, China): triglyceride (TG), total cholesterol (TC), total protein (TP), albumin (Alb), total bile acids (TBA), high-density lipoprotein cholesterol (HDL-C), low-density lipoprotein cholesterol (LDL-C), alkaline phosphatase (AKP), malondialdehyde (MDA), total bilirubin (TBIL), total superoxide dismutase (T-SOD), aspartate aminotransferase (AST), and alanine aminotransferase (ALT).

### 2.2. Animals and Experimental Design

Male Kunming mice (four weeks of age, *n* = 30) were bought from Beijing Vital River Laboratory Animal Technology Co., Ltd. Upon arrival, animals were allowed to acclimate for 7 days under the animal facility: temperature is 22 ± 2 °C, relative humidity ranging from 55% to 65% and a 12 h cycle of day and night. Execution of animal experimentation adhered to ethical protocols sanctioned by the Shandong Normal University Committee (No. AEECSDNU2024117), with provision of standard diet and ad libitum tap water access throughout the experimental duration.

A total of 30 male mice were randomly allocated into 5 experimental groups, with 6 independent biological replicates in each group: healthy chow group (HC), alcohol model group (ACL) and POA groups. Prior sample size estimation via G*Power (v3.1.9.7) software, utilizing pre-experimental and literature-derived parameters comprising a significance level (α) of 0.05, statistical power of 80%, and expected effect size of 1.8, yielding a calculated cohort size of 5 subjects per group. Adjustment for an anticipated attrition rate of approximately 20% resulting in the final inclusion of 6 mice per group, a protocol adhering to ARRIVE 2.0 guidelines and the 3R principles of animal ethics. The POA groups were divided into three groups with different doses: 50 (POAL), 100 (POAM) and 150 (POAH) mg/kg. Mice of the HC group received normal drinking water ad libitum, whereas those in the remaining four groups were intragastrically administered 0.4 mL of 53% (4–5.5 g/kg/day) ethanol once daily. Under our experimental conditions, the maximum safe gavage volume for intragastric administration in Kunming mice was 0.4 mL. Increasing gavage volume raises risk of gastric mechanical injury, and higher alcohol concentration causes severe gastric mucosal damage. Therefore, this model used fixed-volume alcohol gavage instead of strictly body weight-adjusted ethanol dosage. POA was delivered via intragastric gavage, the same administration route as alcohol. The vehicle for POA solution was distilled water. POA gavage was performed synchronously with alcohol gavage every day throughout the full 9-week modeling period after alcohol administration. The weight of all experimental animals was monitored daily throughout the experiment. Animals were anesthetized and used cervical dislocation to euthanize at the experimental endpoint, and the blood was obtained from heart of mice. The collected blood was centrifuged (3000 r/min, 4 °C, 10 min), after which the supernatant was extracted and stored at −80 °C. In addition, various tissue samples—including liver, spleen, stomach, kidney, epididymal fat, and colonic contents—were harvested and snap-frozen at −80 °C for downstream analyses.

### 2.3. Determination of Serum Clinical Indexes and Hepatic Biomedical Indexes

All biochemical parameters (ALT, AST, TBA, TP, AKP, TBIL, Alb, HDL-C, LDL-C, MDA, T-SOD, TC, and TG) were tested using kits from Nanjing Jiancheng Co., Ltd (Nanjing, China). For hepatic tissue analysis, 0.5 g of liver specimen in 4.5 mL of subzero physiological saline was homogenized using a mechanical homogenizer. This homogenate was centrifuged (3000 rpm, 10 min, 4 °C), and the supernatant was separated then preserved at −80 °C. Hepatic levels of TP, AKP, TBA, Alb, AST, ALT, MDA, and T-SOD were measured using the corresponding kits, whereas the remaining indicators were determined in plasma samples.

### 2.4. Histopathological Observation of Liver Tissues

The harvested liver was immediately placed in 4% paraformaldehyde, and the specimens were dehydrated using a gradient ethanol solution of increasing concentration. The dehydrated tissue was cleared using xylene. Then, the cleared tissue was placed into molten paraffin wax and allowed to solidify, forming a tissue block. Paraffin embedding specimens were sectioned to a thickness of approximately 5 μm. These sections were stained with H&E as well as Oil Red O and observed under an optical microscope to evaluate cell morphology, tissue architecture, and pathological alterations.

### 2.5. 16S rRNA Gene Sequencing for Detecting the Gut Microbiome

Bacterial DNA was extracted from colonic content. The sequencing platform requirements were followed to construct a library from the purified PCR products, including adapter ligation and further PCR amplification. The constructed sequencing library was submitted to the Illumina Novaseq6000 (Illumina, Inc. Hayward, CA, America) platform for sequencing execution [19]. The sequences were classified into different microbial populations or taxonomic units (such as OTUs, operational taxonomic units) based on their similarity to known genes in reference databases, and each sequence was assigned to a specific taxonomic level such as species, genus, or family [20]. Microbial community richness was quantified using the Chao1 and ACE indices, while microbiota diversity was quantified with the Shannon and Simpson indices [21].

The Rarefaction Curve is achieved through the use of the plyr package in R software (v4.5.1). Venn diagram analysis was conducted in R software (v4.5.1) with the ggplot2 package to visualize shared and unique OTUs across experimental groups. β diversity was estimated through PCA, PCoA, and NMDS, with analyses implemented using the ade4 and ggplot2 packages [22]. Species abundance bar charts and phylogenetic tree were drawn in Perl through SVG function. Phylum-level microbial composition was analyzed based on the seven most abundant bacterial phyla. Additional analyses—including heatmap visualization and LEfSe—were carried out using appropriate R software [23].

### 2.6. Metabolomic Profiling by LC-MS

The metabolomic extraction protocol was modified slightly based on a published procedure [24]. Briefly, 100 mg liquid nitrogen-frozen colonic contents was ground; vortex oscillation was carried out in the pre-cooled 80% methanol solution, incubated for 5 min at 0 °C, and then centrifuged (15,000× *g*, 4 °C, 20 min). The supernatant was diluted with 53% methanol, added to a new Eppendorf tube, centrifuged again, then collected for LC-MS untargeted metabolomic analysis.

Untargeted metabolomics was performed on a Vanquish UHPLC (Thermo Fisher, Bremen, Germany) coupled with Q Exactive™ HF Orbitrap mass spectrometer (Thermo Fisher, Germering, Germany). Separation was carried out on a Hypersil Gold column (Thermo Fisher, Runcorn, UK) (100 × 2.1 mm, 1.9 μm) at 0.2 mL/min over a 12 min gradient: hold 2% B (methanol, mobile-phase A: 0.1% formic acid-water) for 1.5 min, 2–85% B (3 min), 85–100% B (10 min); reset to 2% B at 10.1 min; and hold until 12 min.

MS acquisition operated in positive/negative ESI data-dependent acquisition (DDA) mode, *m*/*z* 100–1500. Source parameters: spray voltage 3.5 kV, sheath gas 35 psi, auxiliary gas 10 L/min, capillary temperature 320 °C, S-lens RF 60, auxiliary heater 350 °C.

Metabolite identification adopted three confidence levels: Level 1 (MS1, MS2 and retention-time match), Level 2 (MS1 + MS2 match), Level 3 (MS1-only match). A spectral similarity cutoff of 0.5 (10 ppm mass tolerance) was used for Levels 1–2; metabolites scoring below 0.5 were discarded. Candidate metabolites were annotated against an in-house MS^2^ library.

Pooled QC samples were interspersed throughout the run to monitor instrument stability and assessed via Pearson correlation of metabolite relative quantitation values across QCs (|r| closer to 1 indicates better data quality).

Metabolite annotation was performed by matching mass spectral features to KEGG, HMDB and LIPIDMaps. Multivariate statistical analyses were performed with metaX. Univariate significance was tested via Student’s t-test. Screening of differential metabolites was based on the thresholds: VIP ≥ 1, *p* < 0.05, |log2FC| ≥ 0.58. Volcano plots were drawn with R’s ggplot2 package.

For heatmaps, differential metabolite intensity was normalized via z-score transformation and plotted with R software (v4.5.1). Correlation of differential metabolites was investigated by Pearson method with R’s cor() function, and *p*-values were obtained via cor.mtest(). Significant correlations were determined at *p* < 0.05, and correlation matrices were visualized with R’s corrplot package. KEGG pathways enrichment of differential metabolites were performed against the KEGG database.

### 2.7. Correlation Analysis and Statistical Analysis

Spearman’s correlation analysis was employed to analyze correlation between hepatic injury-related biomarkers, gut microbial taxa, and metabolites, with computations performed in R (v4.5.1). Data presentation as mean ± standard deviation. Performance of ANOVA tests via SPSS 20. Determination of statistical significance at *p* < 0.05. When the overall ANOVA result was statistically significant (*p* < 0.05), the LSD post hoc test was applied for pairwise comparisons between groups.

## 3. Results

### 3.1. Effect of POA Ingestion on Body Weight in AFLD Mice

**Figure 1:**
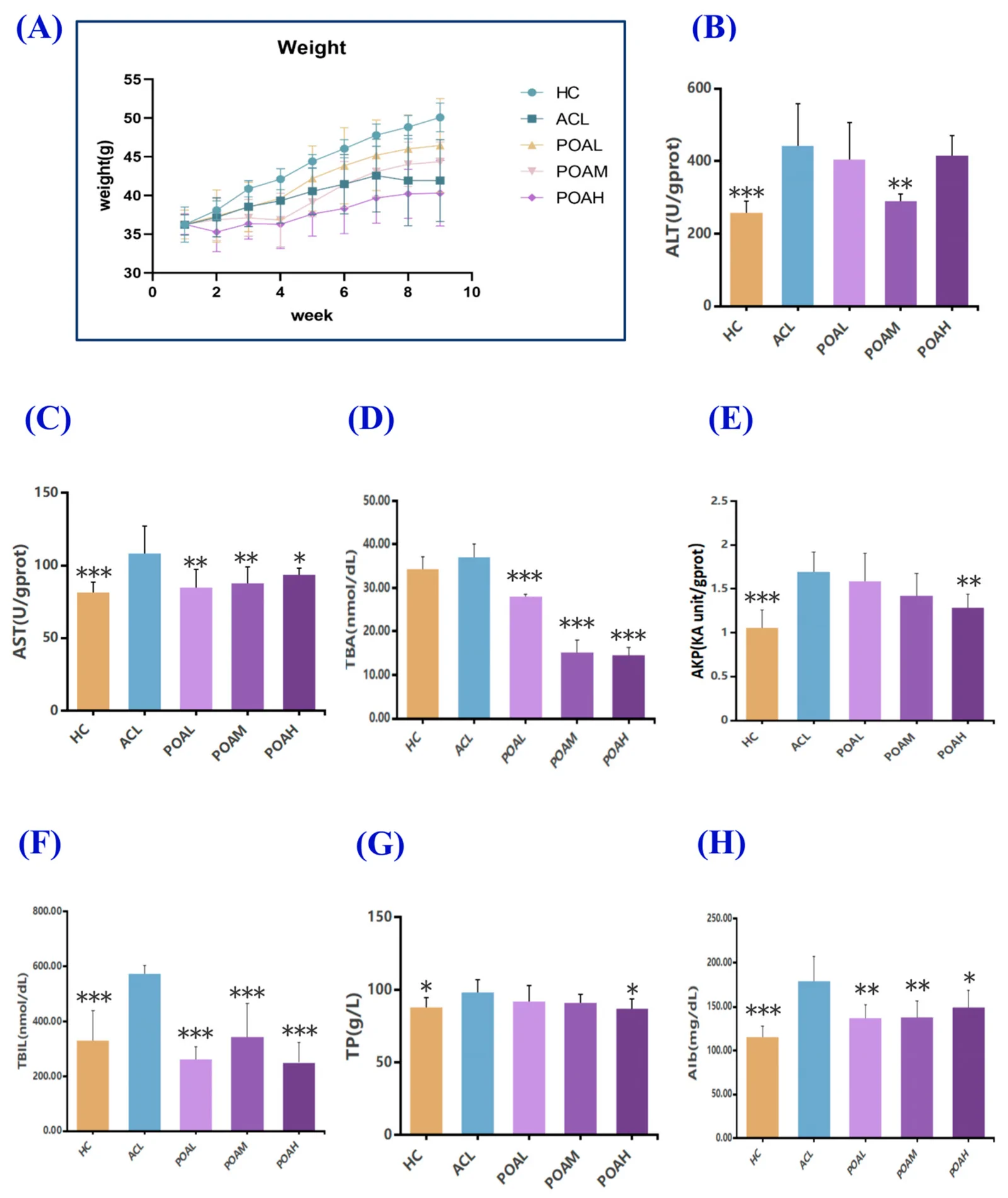
Effects of POA on the weight, protein levels and the liver function of liver in the mice of AFLD-Weight (**A**), ALT (**B**), AST (**C**), TBA (**D**), AKP (**E**), TBIL (**F**), TP (**G**) and Alb (**H**). * *p* < 0.05, ** *p* < 0.01, *** *p* < 0.001 vs. ACL group.

Figure 1A shows the weight change of mice over 9 weeks. The ACL mice showed lower weight growth than the HC mice and the ACL mice began to lose weight in the 7th week. Medium and low doses of POA supplements can effectively alleviate these effects. In contrast, the POAH mice maintained persistently minimum weight throughout the entire experimental period.

### 3.2. Effect of POA Ingestion on Liver Function in AFLD Mice

AST, ALT, AKP, TBA and TBIL levels are key indicators of liver function [25]; when the liver is damaged, the levels will rise. As shown in Figure 1B–F, hepatic concentrations of ALT, AST, TBIL, and AKP were decreased in the HC mice relative to the ACL mice (*p* < 0.001), and TBA levels were also increased. Following POA administration at different doses, the ALT levels decreased, respectively, from 441.47 U/g prot to 404.17 U/g prot, 288.79 U/g prot (*p* < 0.01), and 414.38 U/g prot (Figure 1B), with AST levels dropping, respectively, by 21.7% (*p* < 0.01), 19.1% (*p* < 0.01) and 13.9% (*p* < 0.05) (Figure 1C). TBA levels decreased by 24.3% (*p* < 0.001), 58.9% (*p* < 0.001) and 61.0% (*p* < 0.001) (Figure 1D). AKP levels decreased by 6.4%, 16.4% and 24.1% (*p* < 0.01) (Figure 1E). TBIL levels decreased by 54.1% (*p* < 0.001), 39.9% (*p* < 0.001) and 56.4% (*p* < 0.001) (Figure 1F). The potentiality of POA for the enhancement of hepatic function was identified in murine models of AFLD.

In mice of the ACL group, TP and Alb levels increased, respectively, from 87.96 ± 6.58 g/L and 114.84 ± 13.12 mg/dL in the HC mice to 98.07 ± 8.75 g/L (*p* < 0.05) and 178.61 ± 27.98 mg/dL (*p* < 0.001) (Figure 1G,H). However, as can be seen in Figure 1G,H, these levels diminished when mice were treated with different doses of POA, with TP dropping by 6.1%, 5.7%, and 11.3% (*p* < 0.05), respectively, the Alb levels descended by 23.5% (*p* < 0.01), 22.9% (*p* < 0.01), and 16.7% (*p* < 0.05). TP and Alb are non-specific indicators that are influenced by nutritional status, water intake, plasma volume, and overall condition. They were used as auxiliary indicators to assess liver injury.

### 3.3. Effect of POA Ingestion on Lipid Metabolism and Antioxidant Capacity in AFLD Mice

**Figure 2:**
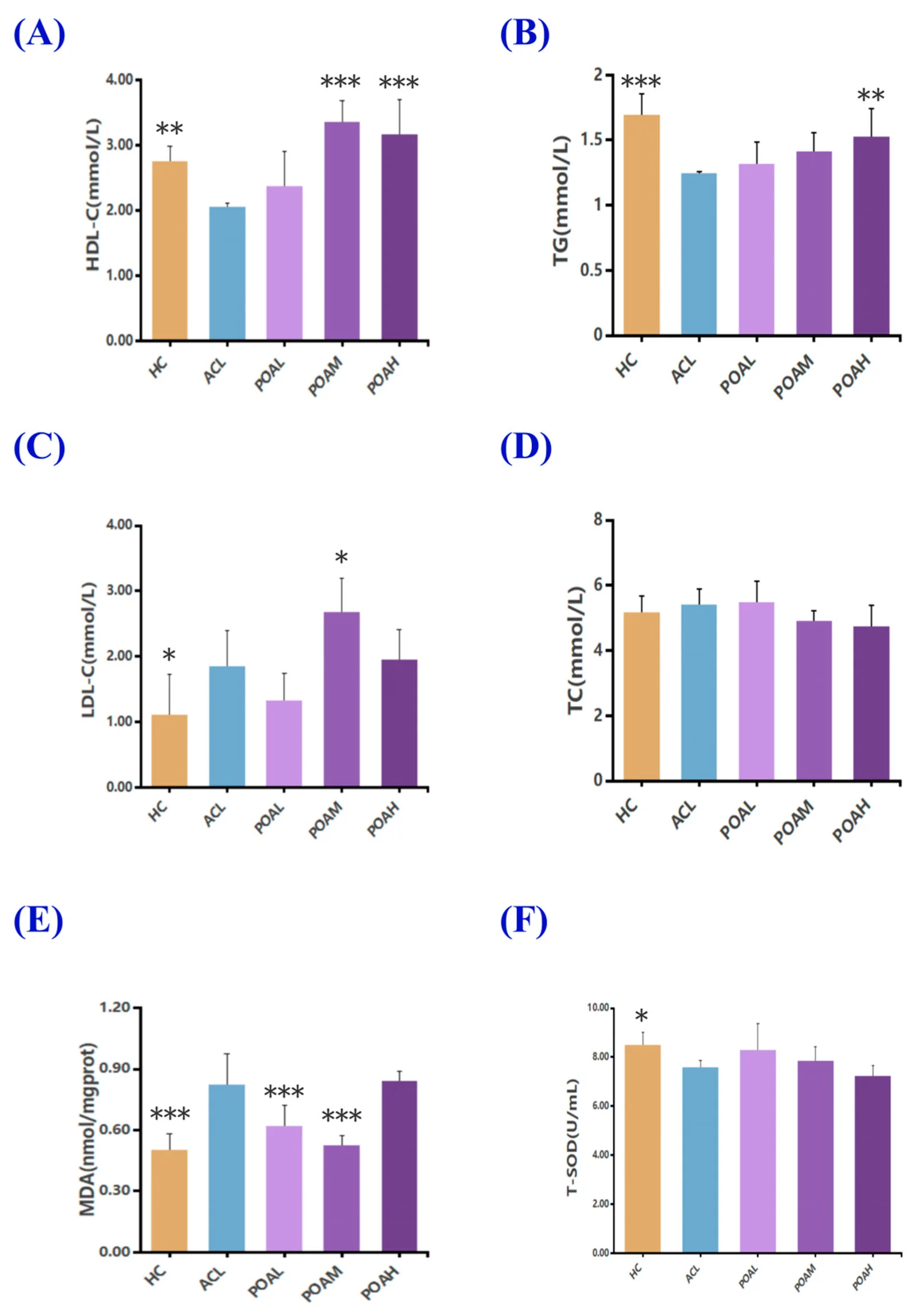
Effects of POA on the antioxidant capacity and the lipid homeostasis in the mice of AFLD-HDL-C (**A**), TG (**B**), LDL-C (**C**), TC (**D**), MDA (**E**), and T-SOD (**F**). * *p* < 0.05, ** *p* < 0.01, *** *p* < 0.001 vs. ACL group.

Hepatic injury is frequently accompanied by disruptions in lipid metabolic homeostasis [26]. Therefore the levels of HDL-C, LDL-C, TC, and TG were measured and shown in Figure 2A–D. The HDL-C and TG levels declined by 25.6% (*p* < 0.01) and 26.5% (*p* < 0.001) in the ACL mice, whereas LDL-C and TC increased by 65.7% and 4.8% (*p* < 0.05, *p* > 0.05). POA supplementation at different doses enhanced HDL-C by 15.3%, 63.3% (*p* < 0.001), and 54.2% (*p* < 0.001) (Figure 2A) and increased TG by 5.9%, 13.8%, and 22.9% (*p* < 0.01) (Figure 2B). In the POAM mice, the levels of LDL-C exhibited a reduction of 44.6% (*p* < 0.05) compared with the ACL mice (Figure 2C). Moreover, the TC levels showed an 8.2% decrease in the POAH mice when relative to the ACL mice (Figure 2D).

Oxidative imbalance is inextricably connected with lipid metabolic homeostasis [27]. Hence the contents of MDA and T-SOD were determined, and these quantitative data were shown in Figure 2E,F. Hepatic MDA concentration in the ACL mice (8.24 ± 1.50 nmol/mL) was higher than that in the HC mice (5.03 ± 0.77 nmol/mL) (*p* < 0.001). T-SOD activity (7.58 ± 0.28 U/mL) was lower than in the HC mice (8.50 ± 0.50 U/mL) (*p* < 0.05). POA administration at different doses reduced MDA levels by 27.4% (*p* < 0.001) and 38.6% (*p* < 0.001), respectively (Figure 2E). The T-SOD level increased from 7.58 ± 0.28 U/mL in the ACL mice to 8.30 ± 1.08 U/mL in the POAL mice (Figure 2F). In aggregate, these results suggest that POA alleviates alcohol-induced hepatic injury by modulating lipid metabolic pathways that are linked to disturbed redox homeostasis.

### 3.4. Effect of POA Ingestion on the Histopathology of Liver Tissues in AFLD Mice

**Figure 3:**
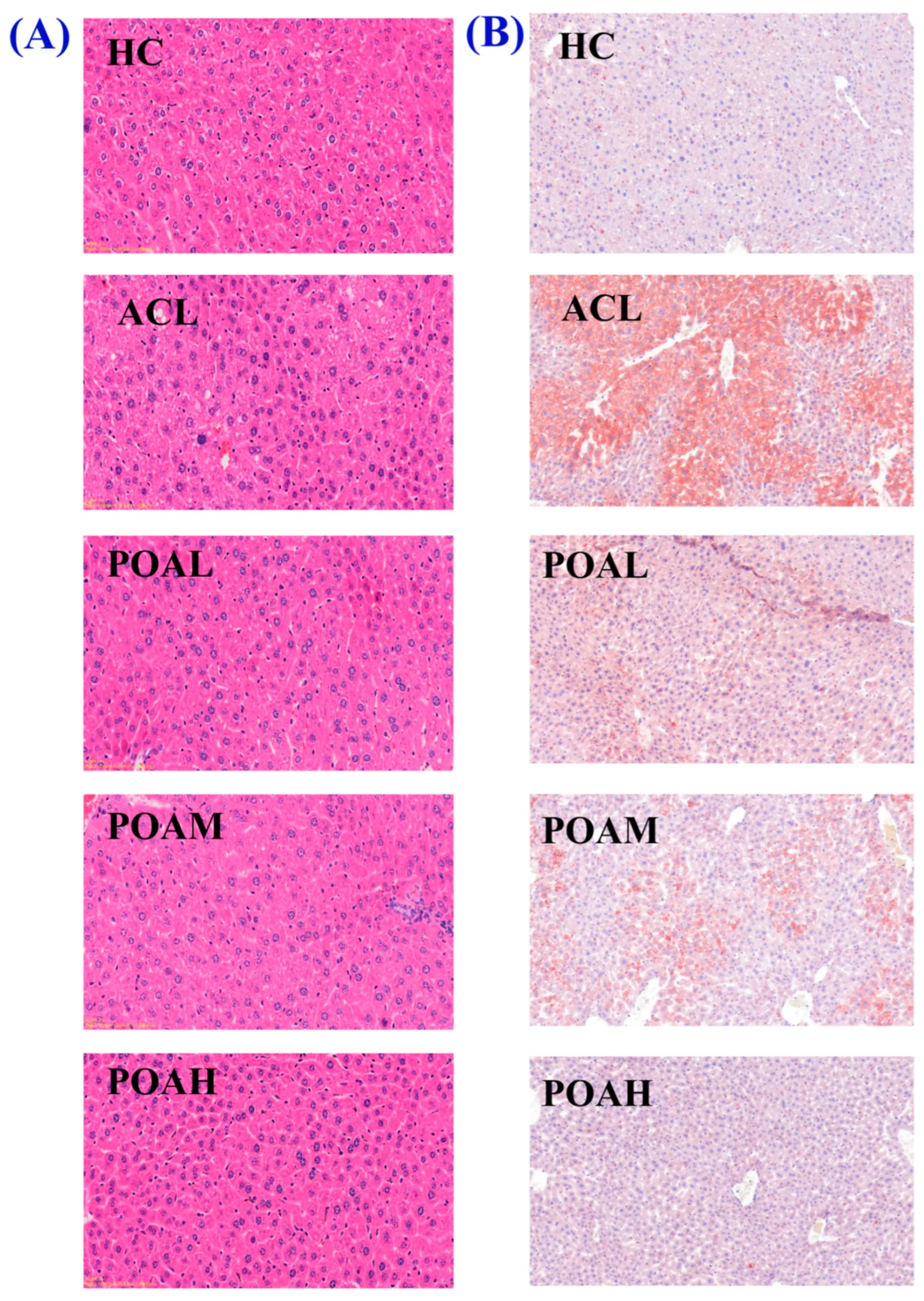
Effects of POA on the histopathological changes in the livers of mice treated with alcohol–liver hepatocytes stained with H&E (**A**) and Oil Red O (**B**).

Direct verification of the counteraction of POA against hepatic toxicity in AFLD murine models, histological and lipid accumulation assessment of liver biopsy slices via H&E and Oil Red O staining, and data presentation in Figure 3A. In the ACL group, the core pathological features of AFLD were clearly observed. Hepatocytes from the ACL mice displayed prominent ballooning degeneration, inflammatory cell infiltration, and indistinct cellular boundaries compared with the HC mice (Figure 3A). POA-treated mice exhibited marked histological improvement, characterized by reduced hepatocellular ballooning and clearer cellular margins. In Figure 3B, liver sections from the ACL mice exhibited substantial lipid droplet accumulation relative to normal liver tissue from the HC mice. After intervention with POA, liver fat accumulation in mice was significantly diminished, particularly in the POAH mice. This may be caused by alcohol metabolism and lipid metabolic disorder, which were restored under the intervention of POA.

### 3.5. Effect of POA Ingestion on the Composition of Gut Microbiota in AFLD Mice

**Figure 4:**
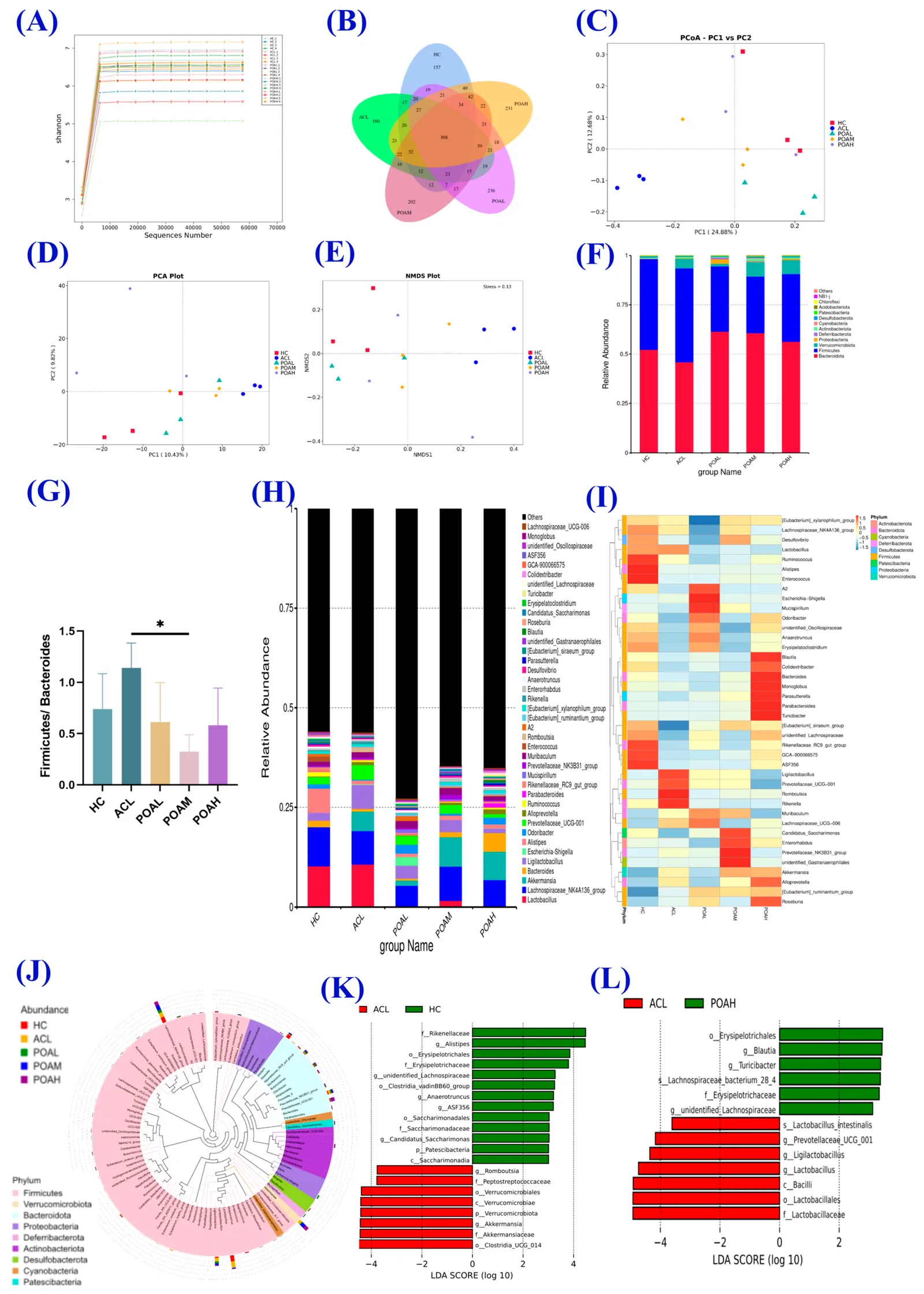
The rarefaction curve analysis (**A**). The Venn diagram (**B**). The PCA plot (**C**). The PCoA plot (**D**). The NMDS clustering plot (**E**). The bar chart of relative species abundance at the phylum level (**F**). The change of *Firmicutes*/*Bacteroidete* (**G**). The relative abundance bar chart of gut microbiota at the genus level in mice of different groups (**H**). The abundance heatmap at the genus level (**I**). The cladogram representative (**J**). LEfSe analysis of gut microbiota in HC group and ACL group mice (**K**). LEfSe analysis of gut microbiota in ACL group and POAH group mice (**L**).

The modulatory role underlying POA-mediated alleviation of alcohol-induced hepatic injury in murine models was elucidated, and an examination of intestinal microbiota distribution was performed. We assured sequencing data volume adequacy for accurate capture of intra-sample species diversity during microbial diversity analysis, executed a rarefaction analysis for the determination of observational sufficiency in obtaining reasonable estimates of sampling-derived quantities, and performed an indirect reflection of sample species richness. We demonstrated the sequencing depth coverage of essentially all sample species (Figure 4A).

Venn diagram analysis was employed to assess shared and exclusive OTUs across the five groups. The total number of OTUs common to all groups is 368. Group-specific exclusive OTUs were quantified as 157, 160, 220, 236, and 231 in the HC, ACL, POAL, POAM, and POAH groups, respectively (Figure 4B). To evaluate between-group differences in microbial community structure, we performed dimensionality reduction analyses, including PCA, PCoA, and NMDS [28]. According to Figure 4C–E, good separation is observed between the groups.

To gain deeper insight into characterizing the modulatory functions of POA on mouse gut microbiota with chronic alcohol exposure, we analyzed microbial composition across diverse taxonomic ranks. At the phylum level, alcohol consumption increased the *Firmicutes/Bacteroides* ratio, with elevated *Firmicutes* abundance and reduced *Bacteroidetes* abundance. POA intervention reversed these alterations, decreasing the *Firmicutes/Bacteroidetes* ratio (*p* < 0.05) while lowering *Firmicutes* and increasing *Bacteroidetes* abundances (Figure 4F–G). At the genus taxonomic level, the abundance of genera such as *Lactobacillus*, *Akkermansia*, *Ligilactobacillus*, *Prevotellaceae UCG-001*, *Alloprevotella*, *Romboutsia*, and *Rikenella* increased in the ACL mice compared with HC mice, and the abundance of *Bacteroides*, *Alistipes*, *Odoribacter*, *Ruminococcus*, and *Rikenellaceae_RC9_gut_group* decreased. After POA intervention the relative proportions of *Ligilactobacillus, Prevotellaceae UCG-001*, *Romboutsia*, and *Rikenella* decreased, and the relative proportions of *Bacteroides, Odoribacter*, and *Rikenellaceae_RC9_gut_group* increased (Figure 4H–I).

We screened for bacteria in the intestinal tract of mice that exhibited significant alterations, and LEfSe analysis was carried out. Figure 4J–L demonstrate the differences of the bacteria between groups. The characteristic bacterial genera in the HC mice were *Alistipes*, *Erysipelotrichales*, *Saccharimonadaceae* and *Patescibacteria*. The ACL mice exhibited characteristics of dysbiosis, among which the abundances of Clostridia UCG 014, *Verrucomicrobiota*, *Akkermansia*, and *Peptostreptococcaceae* were increased (Figure 4K). In the POAH mice, the abundances of *Erysipelotrichales*, *Blautia*, *Turicibacter*, and unidentified *Lachnospiraceae* were increased compared with the ACL mice (Figure 4L).

### 3.6. Effect of POA Ingestion on the Metabolite in AFLD Mice

**Figure 5:**
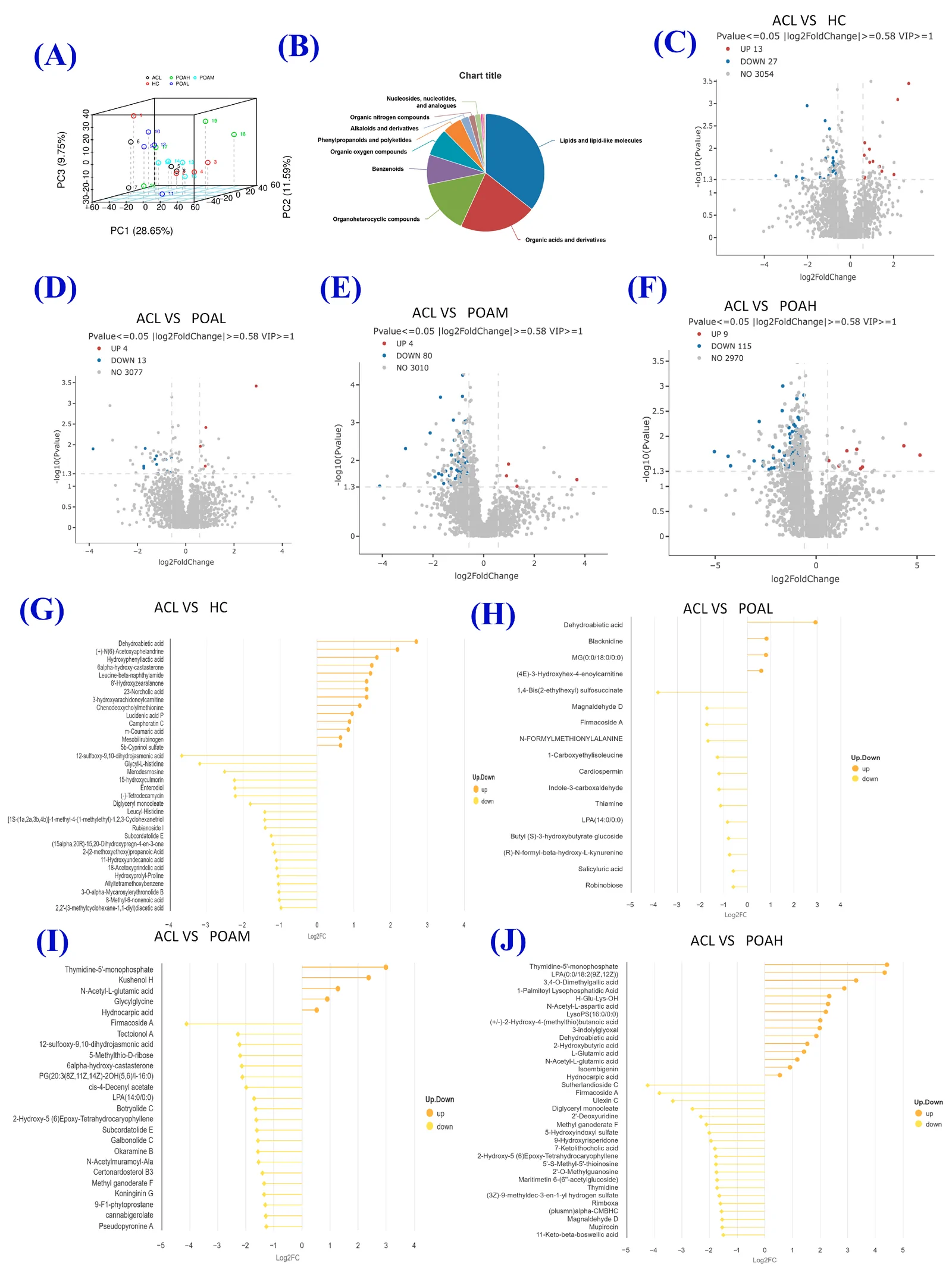
PCA model (**A**). Pie chart of metabolite classification (**B**). Volcano plots (**C**–**F**). Matchstick diagram (**G**–**J**).

Metabolomic analysis of murine models was conducted for the elucidation of POA-mediated attenuation mechanisms in alcohol-induced hepatic injury. Based on the PCA model (Figure 5A), there was a dispersion of data distribution across the five experimental groups (HC, ACL, POAL, POAM, POAH). PCA-derived quantification of total variance attribution to the first three principal components (PC1, PC2, PC3) occurred at 28.65%, 11.59%, and 9.75%, respectively. Figure 5B depicts the proportional distribution of differential metabolite within the sample, encompassing 9 major compound classes, with lipids and lipid-like molecules, organic acids and their derivatives, and organoheterocyclic compounds representing the main categories.

The ACL mice were compared and analyzed pairwise with other groups to identify differential metabolites and conduct pathway enrichment analysis. As illustrated in Figure 5C–F, volcano plots were generated using metabolite peak areas, FC values, and *p*-values. Differential metabolites were identified. Compared with the HC mice, 13 metabolites were upregulated, and 24 metabolites were downregulated in the ACL mice (Figure 5C). Following POA treatment at 50, 100, and 150 mg/kg, the numbers of upregulated metabolites were 4, 4, and 9, respectively, while the numbers of downregulated metabolites were 13, 80, and 115, respectively (Figure 5D–F).

**Figure 6:**
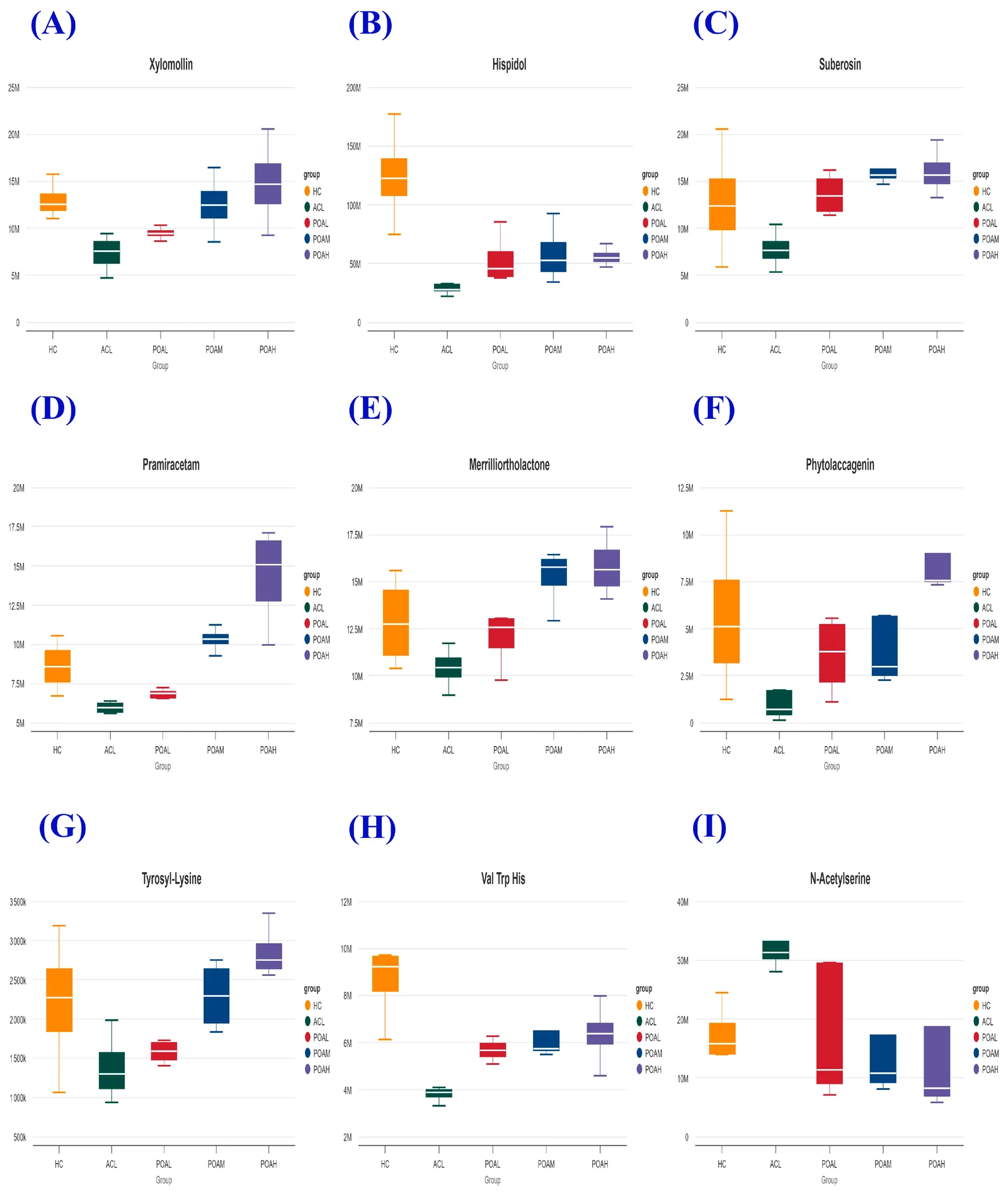
Box plot of Xylomllin (**A**). Box plot of Hispidol (**B**). Box plot of Suberosin (**C**). Box plot of Pramiracetam (**D**). Box plot of Merrilliortholactone (**E**). Box plot of Phytolaccagenin (**F**). Box plot of Tyrosyl-Lysine (**G**). Box plot of Val Trp His (**H**). Box plot of N-Acetylserine (**I**).

Extraction of the top 20 significantly altered metabolites derived from differential metabolite profiles across comparative mice combinations, and the matchstick diagram results were shown in Figure 5G–J. The metabolites of the ACL mice such as Hispidol, Xylomollin, Suberosin, Merrilliortholactone, Phytolaccagenin, Tyrosyl-Lysine and Val-Trp-His were decreased, and *N*-Acetylserine was increased. Under the intervention of POA, the levels of the aforementioned metabolites were restored (Figure 6A–I).

**Figure 7:**
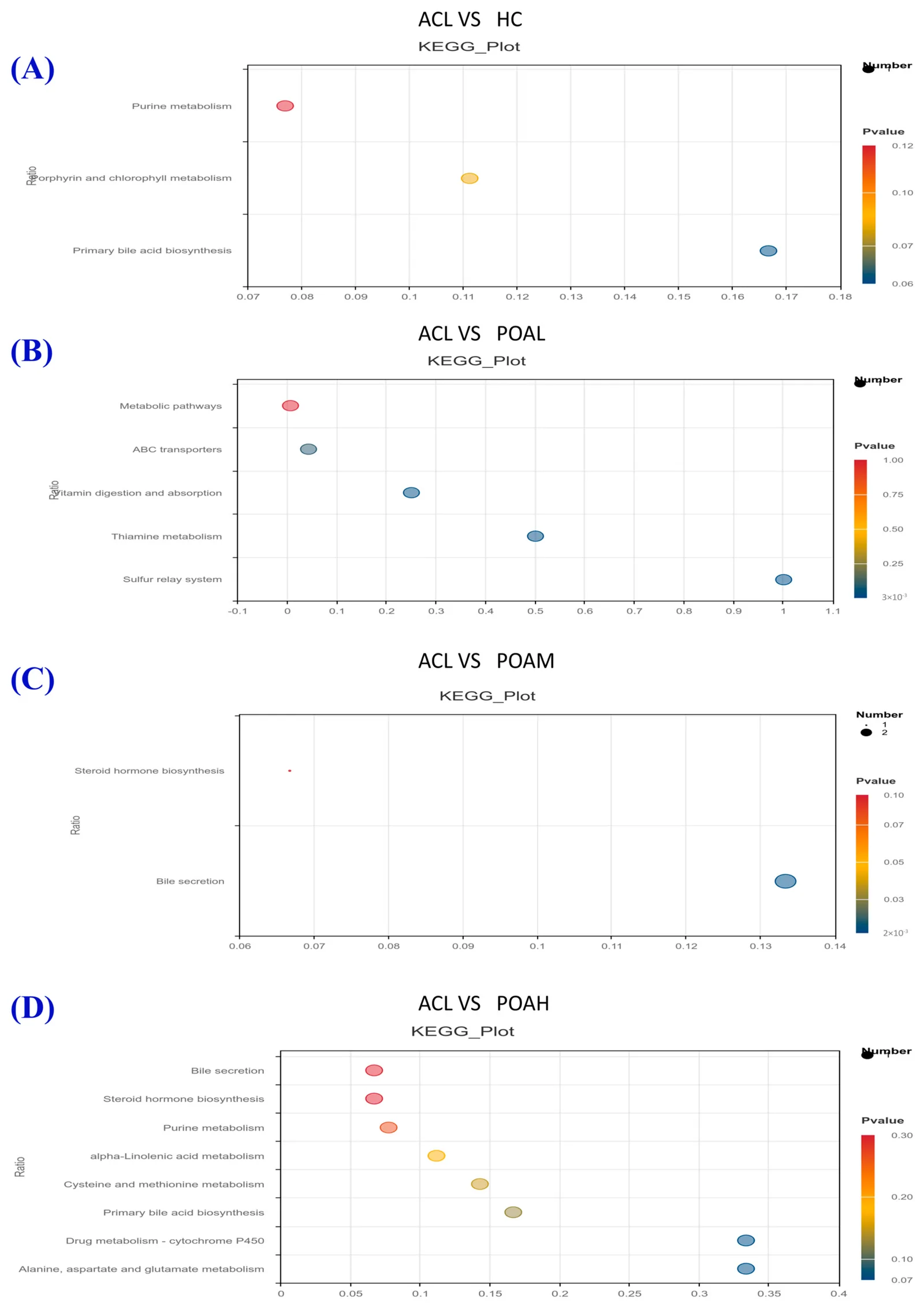
KEGG enrichment bubble plot ACL VS HC based on metabolic pathway analysis (**A**). KEGG enrichment bubble plot ACL VS POAL based on metabolic pathway analysis (**B**). KEGG enrichment bubble plot ACL VS POAM based on metabolic pathway analysis (**C**). KEGG enrichment bubble plot ACL VS POAH based on metabolic pathway analysis (**D**).

As shown in Figure 7, metabolic pathways exhibiting significant divergence between the ACL cohort and comparative groups were identified via KEGG pathway enrichment analysis. Metabolites from the HC versus ACL comparison were mapped onto the KEGG database, yielding three matched metabolic pathways. These pathways include purine metabolism, porphyrin and chlorophyll metabolism, and primary bile acid biosynthesis (Figure 7A). Five correlated KEGG pathways were identified via enrichment analysis comparing the ACL mice and POAL mice, including metabolic pathways, ABC transporters (*p* < 0.05), vitamin digestion and absorption (*p* < 0.05), thiamine metabolism (*p* < 0.01), and the sulfur relay system (*p* < 0.01) (Figure 7B). Two related KEGG pathways were enriched between the ACL mice and POAM mice, including steroid hormone biosynthesis and bile secretion (*p* < 0.01) (Figure 7C), and eight related KEGG pathways were enriched between the ACL mice and POAH mice, including bile secretion; steroid hormone biosynthesis; purine metabolism; alpha-Linolenic acid metabolism; cysteine and methionine metabolism; primary bile acid biosynthesis; drug metabolism-cytochrome P450; and alanine, aspartate and glutamate metabolism (Figure 7D).

In summary, the metabolic pathways most significantly altered between the ACL and HC mice were associated with nucleotide metabolism, cofactor and vitamin metabolism, and lipid metabolism. In contrast, the differential metabolites between the ACL and POAH mice were primarily overrepresented in pathways associated with the digestive system, lipid metabolism, and amino acid metabolism.

### 3.7. Correlation Between Liver Injury and Altered Metabolites

**Figure 8:**
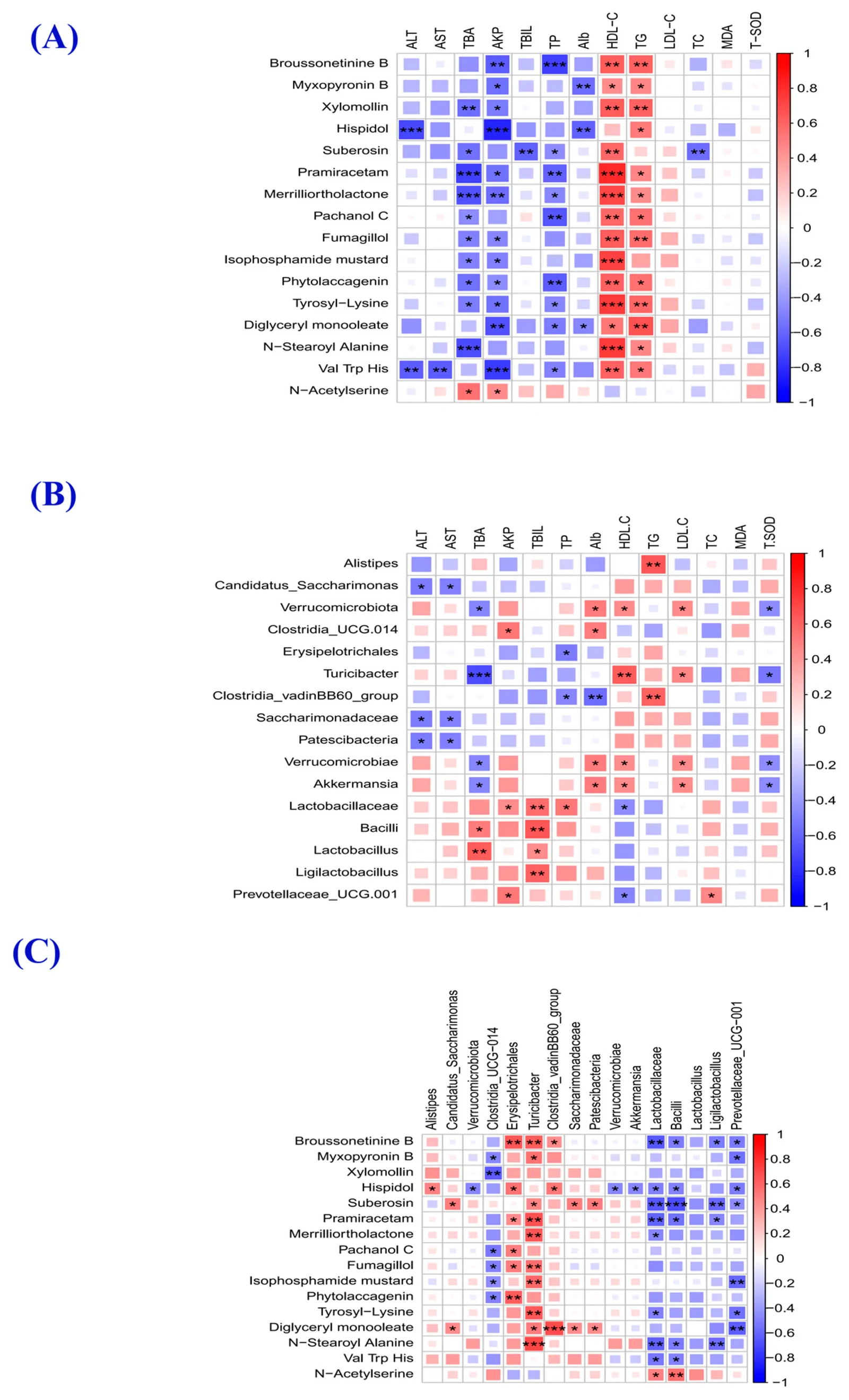
The Spearman correlation heatmap between the liver injury and altered metabolites (**A**), liver injury and intestinal microbiome (**B**) and altered metabolites and intestinal microbiome (**C**). * *p* < 0.05, ** *p* < 0.01, *** *p* < 0.001.

To elucidate the potential correlation between liver injury and significantly altered metabolites, Spearman’s correlation analysis was conducted. Significant correlations between liver injury index (ALT, AST, TBA, AKP, TBIL, TP, Alb, HDL-C, TG, and TC) and altered metabolites (Xylomollin, Hispidol, Suberosin, Merrilliortholactone, Phytolaccagenin, Tyrosyl-Lysine, and Val-Trp-His) were observed in the heatmap (Figure 8A). Strong positive correlations were observed between Xylomollin, Hispidol, Suberosin, Merrilliortholactone, Phytolaccagenin, Tyrosyl-Lysine, Val-Trp-His and HDL-C, TG (*p* < 0.05), while these metabolites exhibited inverse correlations with ALT, AST, TBA, AKP, TBIL, TP, Alb, and TC (*p* < 0.05).

### 3.8. Correlation Between Intestinal Microbiome and Liver Injury

The correlation between hepatic injury and microbiome-associated indices via R language computation was determined for the potential association between liver pathology and the intestinal microbiota. Figure 8B demonstrated that the indicators of liver injury exhibited significant correlations with *Candidatus_Saccharimonas*, *Turicibacter*, *Patescibacteria*, *Akkermansia*, and Prevotellaceae_UCG_001. Among these taxa, *Candidatus_Saccharimonas* and *Patescibacteria* were strongly inversely correlated with ALT, AST, TBA, AKP, TBIL, TC, and MDA but directly correlated with HDL-C, TG, LDL-C, and T-SOD. Conversely, *Turicibacter* and *Akkermansia* were directly correlated with ALT, AST, HDL-C, LDL-C, and MDA but negatively correlated with TBA, TC, and T-SOD. Similarly, Prevotellaceae_UCG_001 also demonstrated a significant positive correlation with ALT, TBA, AKP, TBIL, TP, TC, and T-SOD but were inversely correlated with HDL-C, TG, and LDL-C.

### 3.9. Correlation Between Intestinal Microbiome and Altered Metabolites

Significant associations were demonstrated between intestinal microbiome indicators and altered metabolites (Figure 8C). Significant correlations between altered metabolites (Xylomollin, Hispidol, Suberosin, Val-Trp-His, and *N*-Acetylserine) with microbiome indicators (Clostridia_UCG-014, *Erysipelotrichales*, *Turicibacter*, and Prevotellaceae_UCG_001.) were demonstrated on the heatmap. *Erysipelotrichales* and *Turicibacter* were strongly positively correlated with Xylomollin, Hispidol, Suberosin, Val-Trp-His, *N*-Acetylserine, and other metabolites enriched in the mice (*p* < 0.05). Conversely, Clostridia_UCG-014 and Prevotellaceae_UCG_001 were strongly directly correlated with Xylomollin, Hispidol, Suberosin, Val-Trp-His, *N*-Acetylserine, and other metabolites enriched in the mice (*p* < 0.05). These correlated patterns suggest a potential link between gut microbiota and intestinal metabolic changes.

## 4. Conclusions

The protective actions of POA on liver injury, gut microbiota and metabolic profile in the AFLD mouse model was first investigated. During the intervention period, the ACL mice gained remarkably less weight than the HC mice, a finding consistent with the typical clinical presentation of alcoholic fatty liver disease [29]. After low and moderate doses of POA intervention, the weight of the mice was alleviated. These results indicate that POA alleviates alcohol-induced changes in mouse body weight. However, the weight of the POAH mice further decreased, which is consistent with the findings related to the weight-loss effects of POA [30]. Additionally, liver injury markers (AST and ALT) were remarkably elevated in the ACL mice, consistent with observations in other mouse models of AFLD. POA administration effectively reduced these serum markers, and similar trends were observed for TBA, AKP, and TBIL. Collectively, these results indicate that POA can ameliorate liver damage associated with AFLD.

The levels of TP and Alb in the ACL group increased, and this phenomenon was reversed after the POA intervention. Although TP and Alb cannot be regarded as direct indicators of liver function improvement, they can reflect the changes in homeostasis within the mice. It was found that the intake of POA induced a return of HDL-C and TG, while a decrease in LDL-C and TC improved lipid metabolic disorder in mice with AFLD. Oxidative stress-induced lipid peroxidation is associated with inflammation [31]. Consistent with expectations, MDA levels was markedly greater in the ACL mice, while the T-SOD levels were significantly reduced. After POA intervention, the levels of MDA showed significant recovery, but the level of T-SOD did not exhibit significant improvement. Similarly, the liver H&E and Oil Red O staining conditions further validated the liver injury condition in ACL mice and the protective effect of POA.

The gut microbiota represents a complex and highly multifaceted microbial community that regulates numerous host physiological functions and exerts a crucial effect in maintaining host health [32]. The microbiota in the intestines of humans partake in diverse vital physiological activities within the organism, and compositional perturbations of these microbial communities are associated with the development of multiple diseases [33,34]. Integrated analysis on the basis of 16S rRNA high-throughput sequencing demonstrated that chronic alcohol intake induces intestinal dysbiosis in mice, and intestinal dysbiosis induced by alcohol consumption can be restored by POA. The sparsity curve indicates that the sequencing data are sufficiently reliable. Beta diversity analyses (PCoA, PCA, and NMDS) revealed distinct separation of microbial community structure across the HC, ACL, POAL, POAM, and POAH groups. The results from the heatmap analysis and LEfSe analysis revealed that the abundances of *Clostridia UCG 014*, *Verrucomicrobiota, Akkermansia,* and *Peptostreptococcaceae* had a significant increase within the ACL mice. Meanwhile, the abundance of *Bacteroides*, *Alistipes*, *Odoribacter*, *Ruminococcus,* and *Rikenellaceae_RC9_gut_group* decreased. Abundances were restored to normative levels subsequent to POA administration. Specifically, previous studies have confirmed that the *Verrucomicrobiota* is associated with liver damage, inflammatory responses, and lipid metabolism disorders [35]. Furthermore, the enrichment of *Peptostreptococcaceae* gives rise to obesity and the amelioration of metabolic disorders [36]. Conversely, *Bacteroides*, *Alistipes*, *Odoribacter*, *Ruminococcus,* and *Rikenellaceae_RC9_gut_group* are considered beneficial gut bacteria that may exert hepatoprotective effects via the interplay between the gut and liver. *Bacteroides* has been independently correlated with the progression of non-alcoholic hepatitis, and *Ruminococcus* correlates with hepatic fibrosis [37]. *Alistipes* can alleviate liver fibrosis and inflammation and enhance the body’s antioxidant defense [38]. *Odoribacter* generates short-chain fatty acids such as butyrate, promotes barrier integrity and exerts anti-inflammatory effects [39]. *Rikenellaceae_RC9_gut_group* also promotes intestinal mucosal function and reduces inflammation via short-chain fatty acid production [40]. Beyond that, *Akkermansia* was regarded by most researchers as a new type of potential probiotic. However, it was also demonstrated to be enriched in a mouse model of AFLD [41].

It is widely recognized that ingested dietary components undergo metabolism via the gut microbiota to generate bioactive small molecules, which are subsequently assimilated by the host and exert profound influences on numerous critical physiological processes [42]. To explore whether the hepatoprotective effect of POA is linked to intestinal microbiota and their metabolic products in AFLD mice, we performed LC-MS-based metabolomic profiling of colonic contents. The metabolites in the HC mice, ACL mice and POA mice display distinct clustering patterns. This indicates that alcohol and POA have a significant impact on the metabolites in mice. Furthermore, the detected metabolites were classified and summarized. Predominant metabolite classes, encompassing lipids and lipid-like molecules, organic acids and derivatives, organoheterocyclic compounds, and other significant biomolecular categories, were classified. This finding was in accordance with the aforementioned outcomes regarding lipid metabolic disorder in mice. To further investigate the metabolites, we used volcano plots to screen for differential metabolites between pairwise comparisons. Then, based on the comparison of each group’s differences, the matchstick diagram was drawn to represent the identified differential metabolites. Logarithmic transformation of differential metabolite Fold Change values to base 2 facilitated the selection of the top and bottom 20 ranked entities for visualization. In the ACL mice, Hispidol, Suberosin and Val-Trp-His were decreased but were restored after POA intervention. The four prioritized metabolites identified in this study (Hispidol, Suberosin, Val-Trp-His, and *N*-acetylserine) exert critical regulatory functions in key pathophysiological processes, including lipid metabolism, inflammatory response, hepatic injury, and antioxidant defense. Specifically, Hispidol has anti-inflammatory effects while concurrently enhancing the activity of endogenous antioxidant enzymes, thereby attenuating hepatic lipid accumulation and liver injury [43]. Suberosin also possesses anti-inflammatory and antioxidant properties, which in turn indirectly ameliorates hepatic lipid homeostasis and suppresses liver fibrosis [44]. As for the tripeptide Val-Trp-His, it regulates lipid metabolism through its valine residue and inhibits inflammatory response via its histidine moiety, and it can also enhance the body’s antioxidant capacity [45]. Finally, liver metabolic disorders can induce an increase in *N*-acetylserine levels within the liver, accompanied by severe imbalances in the methionine cycle and one-carbon metabolism [46]. KEGG pathway enrichment analysis of metabolites, revealing the significantly altered metabolic pathways between the ACL and HC mice as primarily involving nucleotide metabolism, cofactor and vitamin metabolism, and lipid metabolism. The significantly different metabolites between the ACL and POA mice are predominantly associated with the digestive system, lipid metabolism, and amino acid metabolism.

## 5. Discussion

Further investigation of potential associations among intestinal liver injury, microbiota composition, and metabolites via additional Spearman’s correlation analysis is required. Metabolites, including Xylomollin, Hispidol, Suberosin, Merrilliortholactone, Phytolaccagenin, Tyrosyl-Lysine and Val-Trp-His, were positively associated with HDL-C and TG but inversely associated with ALT, AST, TBA, AKP, TBIL, TP, Alb, and TC. Similarly, *Candidatus_Saccharimonas* and *Patescibacteria* were inversely associated with ALT, AST, TBA, AKP, TBIL, TC, and MDA but positively associated with HDL-C, TG, LDL-C, and T-SOD. Conversely, *Prevotellaceae_UCG_001* exhibited significant positive associations with ALT, AST, HDL-C, LDL-C and MDA but showed inverse correlations with TBA, TC and T-SOD. *Turicibacter* and *Akkermansia* have a positive associations with ALT, TBA, AKP, TBIL, TP, TC, and T-SOD and inverse associations with HDL-C, TG, and LDL-C. Finally, the five prioritized differential metabolites (xylomollin, hispidol, suberosin, Val-Trp-His, and *N*-Acetylserine) exhibited robust correlations with four bacterial taxa: *Clostridia UCG-014*, *Erysipelotrichales*, *Turicibacter* and *Prevotellaceae UCG-001*. Specifically, *Erysipelotrichales* and *Turicibacter* were strongly positively associated with the above five metabolites as well as other metabolites enriched in the corresponding mouse groups, with statistical significance. Meanwhile, *Clostridia UCG-014* and *Prevotellaceae UCG-001*, which were overrepresented in the ACL mice, also exhibited positive associations with xylomollin, hispidol, suberosin, Val-Trp-His, and *N*-Acetylserine.

Collectively, the findings of this study provide novel insights into the association between gut microbiota, intestinal metabolites and the hepatoprotective effect of POA against AFLD, with three prominent scientific contributions. First, this study systematically investigates and demonstrates the alleviating effect of POA on chronic alcohol-induced mice with AFLD, which expands the biological application scope of ω-7 monounsaturated fatty acids in liver diseases. Our study confirms that POA can simultaneously improve multiple core pathological links of AFLD, including liver function damage, lipid metabolism disorder, and oxidative stress. Second, this study is the first to characterize the impacts of POA on intestinal microbiota and metabolic profiles in the context of AFLD and identifies significant associations between gut microbiota structural shifts, intestinal metabolic profile restoration, and the alleviation of liver injury. These findings provide a new theoretical framework for understanding the biological effects of POA. Third, we identified several key bacterial genera (*Turicibacter*, *Bacteroides*, *Akkermansia*) and core differential metabolites (hispidol, suberosin, Val-Trp-His and *N*-Acetylserine) as potential targets for POA against AFLD, which provides new candidate biomarkers and intervention targets for clinical management of AFLD, but their causal roles require further validation. This study has several limitations. First, no isocaloric control group was included, so the potential influence of caloric differences on the observed metabolic effects cannot be fully excluded. Second, daily food intake was not systematically monitored across groups. Third, liver histological assessment was only qualitative, without quantitative morphometric analysis. Fourth, hepatic triglyceride content was not directly quantified, and only serum lipid profiles were measured. Finally, this study adopted an observational correlational design, which cannot establish causal relationships between gut microbiota/metabolites and hepatic phenotypes; further interventional experiments are required for validation.

## Data Availability

The raw data supporting the conclusions of this article will be made available by the authors on request.

## Abbreviations

POA	Palmitoleic acid
AST	Aspartate aminotransferase
ALT	Alanine aminotransferase
AKP	Alkaline phosphatase
TP	Total protein
Alb	Albumin
TBA	Total bile acid
TC	Total cholesterol
TG	Triglyceride
HDL-C	High-density lipoprotein cholesterol
LDL-C	Low-density lipoprotein cholesterol
T-SOD	Total superoxide dismutase
TBIL	Total bilirubin
MDA	malondialdehyde
H&E	Hematoxylin & eosin
